# Beschleunigte muskuloskeletale Magnetresonanztomographie mit Deep-Learning-gestützter Bildrekonstruktion bei 0,55 T–3 T

**DOI:** 10.1007/s00117-024-01325-w

**Published:** 2024-06-12

**Authors:** Jan Vosshenrich, Jan Fritz

**Affiliations:** 1 Department of Radiology, Grossman School of Medicine, New York University, 660 First Avenue, 10016 New York, NY USA; 2 Klinik für Radiologie und Nuklearmedizin, Universitätsspital Basel, Petersgraben 4, 4031 Basel, Schweiz

**Keywords:** Muskuloskeletale Bildgebung, Beschleunigung, Bildrekonstruktion, Signal-zu-Rausch-Verhältnis, Compressed Sensing, Musculoskeletal imaging, Acceleration, Image reconstruction, Artificial intelligence, Compressed sensing

## Abstract

**Klinisches/methodisches Problem:**

Die Magnetresonanztomographie (MRT) ist ein zentraler Bestandteil der muskuloskeletalen Diagnostik. Lange Akquisitionszeiten können jedoch zu Einschränkungen in der klinischen Praxis führen.

**Radiologische Standardverfahren:**

Die MRT hat sich aufgrund des hohen Auflösungsvermögens und Signal-zu-Rausch-Verhältnisses (SNR) sowie des exzellenten Weichteilkontrastes als Modalität der Wahl in der Diagnostik von Verletzungen und Erkrankungen des muskuloskeletalen Systems etabliert.

**Methodische Innovationen:**

Kontinuierliche Weiterentwicklungen in der Hard- und Softwaretechnologie haben eine bildqualitäts- und genauigkeitsneutrale Beschleunigung von 2D-Turbo-Spin-Echo(TSE)-Sequenzen um den Faktor 4 ermöglicht. Kürzlich vorgestellte, auf Deep Learning (DL) basierende Bildrekonstruktionsalgorithmen helfen, die Abhängigkeit zwischen SNR, räumlicher Auflösung und Akquisitionszeit weiter zu minimieren und erlauben die Anwendung höherer Beschleunigungsfaktoren.

**Leistungsfähigkeit:**

Die kombinierte Anwendung fortschrittlicher Beschleunigungstechniken und DL-basierter Bildrekonstruktion birgt enormes Potenzial, um die Effizienz, den Patientenkomfort und die Zugänglichkeit der muskuloskeletalen MRT bei gleichbleibend hoher diagnostischer Genauigkeit zu maximieren.

**Bewertung:**

DL-rekonstruierte beschleunigte MRT-Untersuchungen haben ihre Praxisreife und ihren Mehrwert innerhalb kürzester Zeit unter Beweis gestellt. Aktuelle wissenschaftliche Erkenntnisse legen nahe, dass das Potenzial dieser Technologie noch nicht ausgeschöpft ist.

**Empfehlung für die Praxis:**

Beschleunigte MRT-Untersuchungen mit DL-gestützter Bildrekonstruktion können zuverlässig in der Primärdiagnostik und Verlaufskontrolle muskuloskeletaler Fragestellungen eingesetzt werden.

## Abstract (en)

**Clinical/methodical issue:**

Magnetic resonance imaging (MRI) is a central component of musculoskeletal imaging. However, long image acquisition times can pose practical barriers in clinical practice.

**Standard radiological methods:**

MRI is the established modality of choice in the diagnostic workup of injuries and diseases of the musculoskeletal system due to its high spatial resolution, excellent signal-to-noise ratio (SNR), and unparalleled soft tissue contrast.

**Methodological innovations:**

Continuous advances in hardware and software technology over the last few decades have enabled four-fold acceleration of 2D turbo-spin-echo (TSE) without compromising image quality or diagnostic performance. The recent clinical introduction of deep learning (DL)-based image reconstruction algorithms helps to minimize further the interdependency between SNR, spatial resolution and image acquisition time and allows the use of higher acceleration factors.

**Performance:**

The combined use of advanced acceleration techniques and DL-based image reconstruction holds enormous potential to maximize efficiency, patient comfort, access, and value of musculoskeletal MRI while maintaining excellent diagnostic accuracy.

**Achievements:**

Accelerated MRI with DL-based image reconstruction has rapidly found its way into clinical practice and proven to be of added value. Furthermore, recent investigations suggest that the potential of this technology does not yet appear to be fully harvested.

**Practical recommendations:**

Deep learning-reconstructed fast musculoskeletal MRI examinations can be reliably used for diagnostic work-up and follow-up of musculoskeletal pathologies in clinical practice.

Die Magnetresonanztomographie (MRT) hat einen beispiellosen technologischen Fortschritt erlebt und sich in der muskuloskeletalen Diagnostik als Methode der Wahl für ein breites Spektrum akuter und chronischer Erkrankungen etabliert. Technisch bedingt lange Untersuchungszeiten können durch moderne Beschleunigungstechniken und neue Deep-Learning-basierte Algorithmen zur Bildrekonstruktion ohne Qualitätsverlust drastisch verkürzt werden. Die technischen Konzepte, ihre Nutzung und der eingeläutete Paradigmenwechsel in der muskuloskeletalen Bildgebung werden in diesem Artikel näher beleuchtet.

## Beschleunigungstechniken

Homogenere statische Magnetfelder, leistungsstärkere Gradientensysteme, ausgefeilte Sende- und Empfangsspulen und kontinuierlich weiterentwickelte Turbo-Spin-Echo(TSE)-Sequenzen haben die Signalausbeute moderner MRT-Geräte erheblich verbessert. Der resultierende Signalüberschuss ermöglicht die Anwendung optimierter Pulssequenzen und höherer Beschleunigungsfaktoren, denn erst durch die beschleunigte Bildakquisition kann das gesamte Potenzial DL-basierter Rekonstruktionsalgorithmen ausgeschöpft werden. Die meisten dieser Algorithmen nutzen existierende Undersamplingkonzepte aus, mit dem Ziel, diagnostische MRT-Bilder basierend aus einer geringeren Menge k‑Raum-Daten zu rekonstruieren.

Insbesondere die parallele Bildgebung, die simultane Mehrschicht-Akquisition und Compressed Sensing (CS) sind einzeln oder in Kombination ideal für die Beschleunigung muskuloskeletaler MRT-Untersuchungen geeignet. Gleichzeitig sollten jedoch Basisparameter wie Echoabstand und Echozuglänge optimiert werden, um Zeitersparnisse zu maximieren.

### Parallele Bildgebung

Die parallele Bildgebung ist auf nahezu jedem MRT-Gerät verfügbar und kann für 2D- und 3D-Bildgebung angewendet werden. Sie reduziert das Auslesen von MR-Signalen auf jede zweite (zweifache Beschleunigung) oder dritte (dreifache Beschleunigung) k‑Raum-Zeile in Phasenkodierrichtung, wenn ortskodierte MR-Signale gleichzeitig von mehreren Elementen einer Phased-Array-Spule aufgenommen werden. 3D-Sequenzen erlauben höhere Beschleunigungsfaktoren durch simultanes Undersampling in Phasen- und Schichtkodierrichtung.

Der physikalische Zusammenhang zwischen Beschleunigungsfaktor (R) und Signal-zu-Rausch-Verhältnis setzt der parallelen Bildgebung allerdings Grenzen. Dieses verringert sich proportional um: $\mathrm{SNR}_{PI}=\frac{SNR}{g\times \sqrt{R}}$, wobei der Geometriefaktor (g) die Heterogenität der Rauschverteilung über das Bild beeinflusst. Eine Reduktion der Akquisitionszeit auf die Hälfte (R = 2) senkt das SNR somit bereits um ca. 30 %. Ein intrinsisch hohes SNR von Turbo-Spin-Echo(TSE)-Sequenzen erlaubt bei 1,5 T und 3 T dennoch eine zweifache (2D-TSE) bzw. vierfache Beschleunigung (3D-TSE) ohne subjektive Qualitätseinbußen oder reduzierte diagnostische Genauigkeit [1, 6, 11, 24].

Bei der 0,55-T-Niederfeld-MRT ergibt sich hingegen ein physikalisches Dilemma. Hier müssen häufig mehr Messungen mit langen Akquisitionszeiten aufgewendet werden, um das gegenüber 1,5 T um ca. 40 % niedrigere SNR auszugleichen. Eine Beschleunigung wäre daher wünschenswert, würde die Signalzugewinne unter Nutzung konventioneller Bildrekonstruktion jedoch neutralisieren.

### Simultane Mehrschichtakquisition

Im Gegensatz zur herkömmlichen, sukzessiven Akquisition einzelner Partitionen, erlaubt die simultane Mehrschichtbildgebung („simultaneous multislice“, SMS) das gleichzeitige Anregen und Auslesen mehrerer Schichten. Sie setzt ebenfalls die Nutzung von Phased-Array-Spulen für die korrekte räumliche Zuordnung simultan akquirierter Signale voraus. Im Gegensatz zur parallelen Bildgebung ist sie nahezu SNR-neutral, da kein Undersampling in Phasenkodierrichtung erfolgt. SMS ermöglicht kürzere Akquisitionszeiten durch kürzere Repetitionszeiten (TR), mehr Echos pro Echozug und eine Reduktion der *Concatenations* (Verkettungen, die die Verteilung einer Schichtakquisition über mehrere TRs erlaubt). Eine Reduktion der Concatenations wird einer TR-Verkürzung in der klinischen Praxis meist vorgezogen, um die Sequenzgewichtung und Flüssigkeitssensitivität nicht negativ zu beeinflussen [8].

Dank der annähernden SNR-Neutralität kann SMS ideal mit paralleler Bildgebung gekoppelt werden. In Kombination multiplizieren sich die Beschleunigungsfaktoren und ermöglichen für die 2D-Bildgebung peripherer Gelenke eine qualitätsneutrale vierfache Beschleunigung bei gleichbleibender diagnostischer Genauigkeit [4, 7, 12].

Technisch ist die SMS-Beschleunigung bei 0,55 T bis 3 T möglich. Limitationen der Anwendbarkeit liegen in höheren spezifischen Absorptionsraten (SAR) durch die simultane Aussendung mehrerer Radiofrequenzpulse und, je nach Hersteller und Gerätemodell, in der Notwendigkeit kostenpflichtiger Lizenzen.

### Compressed Sensing

Im Gegensatz zur parallelen Bildgebung erfolgt das Undersampling beim Compressed Sensing nicht linear, sondern pseudozufällig. Periphere k‑Raum-Daten werden dabei als redundant bzw. weniger essenziell angesehen, bevorzugt ausgelassen und dann iterativ rekonstruiert [22]. Die Akquisitionszeit reduziert sich proportional zum CS-Faktor. Die Bildrekonstruktion ist allerdings technisch aufwändiger als in der parallelen Bildgebung und benötigt leistungsstarke Prozessoren und Grafikkarten in den Host-Computern der MRT-Geräte.

Durch die iterative Bildrekonstruktion ist der SNR-Verlust geringer als bei paralleler Bildgebung. Beide Techniken können daher in Kombination angewendet werden, um eine 3‑ (2D-TSE) bis 6‑fache Beschleunigung (3D-TSE) ohne subjektiven Qualitätsverlust zu erzielen [9, 10, 17, 21]. Darüber hinaus ist das CS-Auslesemuster ideal für die Beschleunigung moderner Sequenzen zur Metallartefaktreduktion, z. B. Slice-Encoding for Metal Artifact Correction (SEMAC), geeignet. Diese tolerieren eine bis zu 8‑fache Beschleunigung und ermöglichen die artefaktfreie Darstellung von Hüft‑, Knie- und OSG-Prothesen bei 1,5 T und 3 T in Akquisitionszeiten von 4 bis 6 min [16]. Bei 0,55 T ist CS-SEMAC derzeit nicht verfügbar. Durch physikalisch bedingt geringer ausgeprägte Metallartefakte sind hier jedoch grundsätzlich weniger SEMAC-Schritte erforderlich, und in Konsequenz meist auch keine Beschleunigung.

## Deep-Learning-gestützte Bildrekonstruktion

Deep-Learning(DL)-Algorithmen können verschiedene Aufgaben in der Rekonstruktion von MRT-Bildern übernehmen und werden stetig weiterentwickelt. Die Fähigkeiten der ersten kommerziellen Algorithmen beschränkten sich auf Rauschunterdrückung, Anhebung der Kantenschärfe, Glättung und Korrektur von PI-assoziierten Aliasing-Artefakten. Neuere Algorithmen bzw. kombinierte Modelle beinhalten zusätzlich oft Interpolation und Super-Resolution, und korrigieren weitere, z. B. SMS-assoziierte Artefakte [22].

Analog zu konventionellen Rekonstruktionsmethoden in der parallelen Bildgebung, wie Generalized Autocalibrating Partial Parallel Acquisition (GRAPPA) und Sensitivity Encoding (SENSE), können DL-basierte Algorithmen entweder im k‑Raum oder im Bildraum arbeiten [19]. Sie bauen häufig auf bestehende Rekonstruktionskonzepte wie GRAPPA und SENSE auf, was auch die enge Beziehung zu den genannten Beschleunigungstechniken erklärt. Die ausführliche Beschreibung existierender Architekturen und Konzepte neuronaler Netze in der Bildrekonstruktion, einschließlich Vor- und Nachteilen verschiedener Modelle übersteigen allerdings den Rahmen dieses Artikels. Der interessierte Leser findet jedoch u. a. in diesen Übersichtsartikeln weitere Details und relevante Literaturstellen [19, 21].

Aktuell bieten alle großen MRT-Gerätehersteller DL-Algorithmen zur Bildrekonstruktion an, deren Konzepte, Fähigkeiten und Anwendungsgebiete sich jedoch unterscheiden.

### Rauschreduktion

Ein niedriges SNR äußert sich meist als störendes Rauschen und schlechte Darstellung feiner anatomischer Strukturen. Höhere Beschleunigungsfaktoren verstärken diesen Bildeindruck. Aufgrund der Spulengeometrie ist die Rauschintensität bei paralleler Bildgebung allerdings ungleichmäßig über das Bild verteilt und am stärksten an weit von den Spulenelementen entfernten Lokalisationen ausgeprägt (entsprechend des Geometriefaktors). Als Faustregel gilt, dass inhomogene Rauschverteilung und Aliasing-Artefakte verstärkt auftreten, wenn der Beschleunigungsfaktor die Anzahl der Spulenelemente in Phasenkodierrichtung überschreitet.

Konventionelle Rekonstruktionsalgorithmen können heterogenes Rauschen nicht adäquat reduzieren, da sie Rauschfilter gleichmäßig über die gesamte Bildfläche anwenden. Dieses Problem kann bei Deep-Learning-basierten Rekonstruktionsalgorithmen durch die Integration von Rauschkarten und Spulensensitivitätsprofilen überwunden werden.

Kommerziell verfügbare Algorithmen erlauben dem Nutzer, die Stärke der Rauschunterdrückung entsprechend persönlicher Präferenzen auszuwählen, z. B. „niedrig“, „moderat“, und „hoch“. Zu berücksichtigen ist, dass eine zu starke Rauschunterdrückung je nach untersuchter anatomischer Region und Sequenzkontrast zu einer subjektiven und ggf. als störend empfundenen Glättung und Kantenunschärfe führen kann [23]. Zusätzlich kann eine Rauschreduktion in Einzelfällen dazu führen, dass Bewegungsartefakte als verstärkt ausgeprägt wahrgenommen werden, da diese nun mehr hervorstechen [20].

Die Fähigkeiten der DL-basierten Rauschreduktion sind allerdings nicht unbegrenzt. Aktuelle, für die klinische Nutzung zugelassene Algorithmen ermöglichen eine 3‑ bis 4‑fache Beschleunigung mittels paralleler Bildgebung. Höhere PI-Beschleunigungsfaktoren können hingegen nicht mehr vollständig kompensiert werden bzw. erfordern eine Offline-Rekonstruktion. Ihre klinische Anwendung wird erst mit zukünftigen Algorithmusgenerationen realisiert werden können.

### Superresolution

Die räumliche Auflösung ist neben Akquisitionszeit und SNR der dritte limitierende Faktor der MRT-Bildgebung. Eine Erhöhung der Bildmatrix bei gleichbleibender Bildgröße konnte bisher nicht ohne eine verlängerte Akquisitionszeit und ohne ein proportional geringeres SNR erreicht werden („There is no free lunch in MRI“). Eine Vielzahl von Herangehensweisen zur Überwindung dieses physikalischen Zusammenhangs mit DL-Algorithmen wurde in den letzten Jahren evaluiert. Der auf den MRT-Geräten in der Institution der Autoren klinisch verfügbare Algorithmus (Deep Resolve Sharp, Siemens Healthineers, Erlangen, Deutschland) wurde an einer großen Anzahl paariger Datensätze mit geringer und hoher Auflösung trainiert und erlaubt auf diese Weise die Vorhersage von Inhalten in der Peripherie des k‑Raums [3, 22]. Hier sind Frequenzen lokalisiert, die Ortsinformationen wie Details, Kanten und Schärfen enthalten, während Kontrastinformationen im Zentrum des k‑Raums gespeichert sind. Die Kombination des DL-Modells mit den tatsächlich akquirierten Rohdaten einer Sequenz kann so die Matrixgröße um den Faktor zwei in beide Schichtrichtungen erhöhen, ohne die Kontrastinformationen der Untersuchung zu verfälschen [22]. Der genannte Algorithmus ist theoretisch auf MRT-Geräten mit Feldstärken von 0,55 T bis 3 T nutzbar, die Verfügbarkeit variiert jedoch nach Modell und Softwarearchitektur. Allerdings kann auch mit anderen klinisch verfügbaren Algorithmen, die noch keine spezielle Superresolutionskomponente beinhalten, eine nahezu SNR-neutrale Erhöhung der Ortsauflösung durch die Nutzung höherer Beschleunigungsfaktoren und DL-basierter Rauschreduktion realisiert werden. Diese ist dann allerdings lediglich zeitneutral und erlaubt keine bzw. nur eine geringe gleichzeitige Verkürzung der Akquisitionszeit.

## Kombinierte Nutzung in der muskuloskeletalen MRT

Die kombinierte Nutzung fortschrittlicher Beschleunigungstechniken und DL-basierter Bildrekonstruktion ermöglicht eine 4‑ bis 8‑fache Beschleunigung von 2D-Turbo-Spin-Echo-Sequenzen mittels paralleler Bildgebung oder kombinierter PI-SMS-Nutzung. Zahlreiche Studien haben die Gleichwertigkeit derartiger Untersuchungsprotokolle im Vergleich zu nichtbeschleunigten und konventionell rekonstruierten Sequenzen unter Nutzung von Algorithmen und MRT-Geräten verschiedener Hersteller in der Bildgebung peripherer Gelenke und der Wirbelsäule untersucht und bestätigt [2, 13, 14, 18]. Die Akquisitionszeiten konnten auf diese Weise um bis zu 75 %, auf teils unter 5 min gesenkt werden. Auch für die dreifache CS-beschleunigte 3‑T-MRT des Kniegelenks mit DL-Superresolution (DLSR) wurde kürzlich die Gleichwertigkeit der Bildqualität zu konventionell rekonstruierten hochaufgelösten Sequenzen bei gleichzeitiger Verkürzung der Akquisitionszeit um 57 % dargelegt [25].

**Abb. 1:**
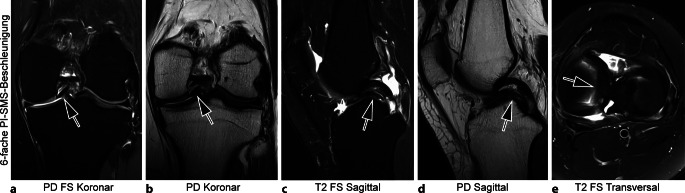
Sechsfach-PI-SMS-beschleunigte 3‑T-MRT (Magnetresonanztomographie, parallele Bildgebung und simultane Mehrschicht Akquisition) des linken Kniegelenks mit Deep-Learning-Superresolution (DLSR; Gesamt-Akquisitionszeit: 04:34 min). 27-jährige Patientin mit Korbhenkelriss des Innenmeniskus (*Pfeile* in **a**–**e**) nach Distorsionstrauma mit „Doppel-HKB-Zeichen“ (*Pfeile* in **c** und **d**). *HKB* hinteres Kreuzband

**Abb. 2:**
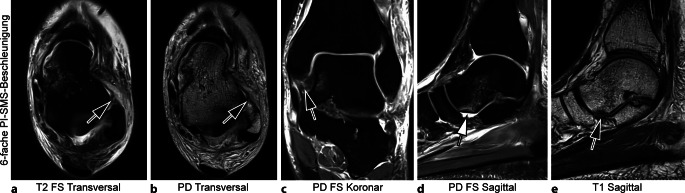
Sechsfach PI-SMS-beschleunigte 3 T MRT (Magnetresonanztomographie, parallele Bildgebung und simultane Mehrschicht Akquisition) des linken Sprunggelenks mit Deep-Learning-Superresolution (DLSR; Gesamt-Akquisitionszeit: 04:48 min). 23-jähriger Patient mit Rupturen des anterioren fibulotalaren Bands (*Pfeile* in **a** und **b**) und der tiefen Anteile des Deltabandkomplexes (*Pfeil* in **c**), sowie Impressionsfraktur des Taluskopfes (*Pfeile* in **d** und **e**), hinweisend auf ein peritalares Subluxationstrauma im Rahmen einer Distorsion

**Abb. 3:**
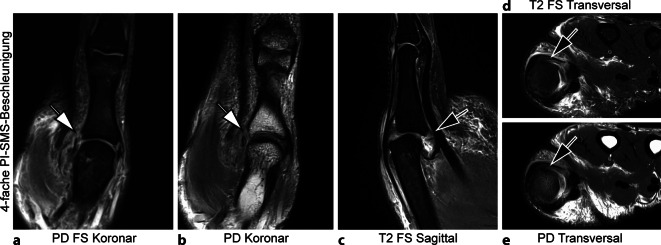
Vierfach PI-SMS-beschleunigte 3 T MRT (Magnetresonanztomographie, parallele Bildgebung und simultane Mehrschicht Akquisition) des rechten Daumens mit Deep-Learning-Superresolution (DLSR; Gesamt-Akquisitionszeit: 06:09 min). 29-jähriger Patient mit vollständiger Ruptur des ulnaren Kollateralbands im Metakarpophalangealgelenk (MCP) I (*Pfeile* in **a**, **b**, **d** und **e**) und begleitender Verletzung der volaren Platte (*Pfeil* in **c**) nach Unfall

**Abb. 4:**
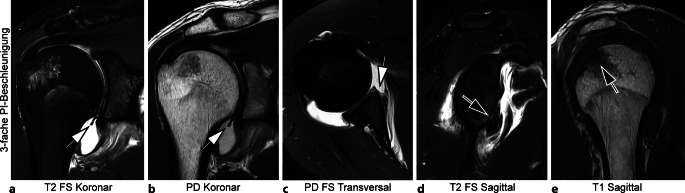
Dreifach PI-beschleunigte 3‑T-MRT (Magnetresonanztomographie, parallele Bildgebung) des rechten Schultergelenks mit Deep-Learning-Superresolution (DLSR; Gesamt-Akquisitionszeit: 06:39 min). 32-jährige Patient mit labraler und osteokartilaginärer Bankart-Läsion (*Pfeile* in **a** bis **d**) und Hill-Sachs-Läsion (*Pfeil* in **e**) nach anteriorer Luxation

Im klinischen Alltag werden derzeit 4‑ bis 6‑fach kombinierte PI-SMS-beschleunigte 3‑T-MRT-Untersuchungen mit DLSR für Rauschreduktion und Superresolution eingesetzt. Die Darstellung von Kniegelenk (Abb. 1), Sprunggelenk (Abb. 2) und Hand bzw. Fingern (Abb. 3) ist so in 4 bis 6 min möglich. Gleichermaßen kann eine 3‑fache PI-Beschleunigung erzielt und die Akquisitionszeit einer MRT-Untersuchung des Schultergelenks (Abb. 4) auf 6 min verkürzt werden. Ausgewählte Untersuchungsprotokolle für die Gelenkbildgebung bei 3 T aus dem Institut der Autoren sind in Tab. 1, 2 und 3 zur Verfügung gestellt. Die Weiterentwicklung klinisch verfügbarer Algorithmen könnte bald eine 10-fache Beschleunigung von 2D-TSE-Sequenzen bei 3 T mit einer Reduktion der Untersuchungszeiten auf 2 bis 3 min Realität werden lassen [5, 22].

**Tab. 1:** Untersuchungsprotokolle für die Gelenkbildgebung bei 3 T aus dem Institut der Autoren

Beschleunigtes 3‑T-MRT-Protokoll des Kniegelenks mit DL-basierter Bildrekonstruktion					
Parameter	Axiale T2-FS	Koronare PD	Koronare PD FS	Sagittale T2 FS	Sagittale PD
Repetitions‑/Echozeit (ms)	3600/57	3700/24	3700/35	3700/56	3700/24
PI-Faktor	3	3	3	3	3
SMS/FOV-Shift-Faktor	2/2	2/2	2/2	2/2	2/2
Echozuglänge	13	13	13	13	13
Receiver Bandbreite (Hz/Pixel)	296	354	301	299	354
Echoabstand (ms)	7,1	8,0	7,1	8,0	8,0
Field of view (mm)	140 × 140	140 × 140	140 × 140	140 × 140	140 × 140
Matrix	272 × 204	336 × 252	272 × 204	304 × 228	336 × 252
Schichtdicke (mm)	3	3	3	3	3
Schichtanzahl	38	36	36	38	38
Phasenkodierrichtung	Rechts-links	Kopf-Fuß	Kopf-Fuß	Kopf-Fuß	Kopf-Fuß
Phasen Oversampling (%)	38	100	100	100	100
Anzahl der Averages	1	1	1	1	1
Flip-Winkel (°)	125	125	125	125	125
Fettsättigung	SPAIR	–	SPAIR	SPAIR	–
Deep Resolve Sharp	On	On	On	On	On
Deep Resolve Boost	High	Medium	High	High	Medium
Akquisitionszeit (mm:ss)	00:43	01:00	00:53	00:57	01:01

*DL* Deep Learning,* PD* protonengewichtet, *PI* parallele Bildgebung, *FS* fettsupprimiert, *SMS* simultane Mehrschicht, *SPAIR* Spectral Attenuated Inversion Recovery (Siemens Healthineers)

**Tab. 2:** Untersuchungsprotokolle für die Gelenkbildgebung bei 3 T aus dem Institut der Autoren

Beschleunigtes 3‑T-MRT-Protokoll des Sprunggelenks mit DL-basierter Bildrekonstruktion					
Parameter	Axiale T2 FS	Axiale PD	Sagittale PD FS	Koronare PDFS	Sagittale T1
Repetitions‑/Echozeit (ms)	4180/39	3800/19	5820/37	4000/39	400/8,9
PI-Faktor	3	3	2	3	3
SMS/FOV-Shift-Faktor	2/2	2/2	2/4	2/2	2/2
Echozuglänge	14	14	16	14	4
Receiver Bandbreite (Hz/Pixel)	305	354	304	305	347
Echoabstand (ms)	9,7	9,6	9,2	9,7	8,9
Field of view (mm)	140 × 100	140 × 143	140 × 140	140 × 103	140 × 140
Matrix	304 × 228	336 × 252	336 × 252	304 × 228	272 × 204
Schichtdicke (mm)	3	3	3	3	3
Schichtanzahl	36	36	28	34	28
Phasenkodierrichtung	Rechts-links	Rechts-links	Anterior-posterior	Rechts-links	Anterior-posterior
Phasen Oversampling (%)	100	50	100	100	100
Anzahl der Averages	1	1	1	1	1
Flip-Winkel (°)	138	121	160	126	127
Fettsättigung	SPAIR	–	SPAIR	SPAIR	–
Deep Resolve Sharp	On	On	On	On	On
Deep Resolve Boost	High	Medium	High	High	Medium
Akquisitionszeit (mm:ss)	00:47	00:51	01:46	00:49	00:35

*DL* Deep Learning,* PD* protonengewichtet, *PI* parallele Bildgebung, *FS* fettsupprimiert, *SMS* simultane Mehrschicht, *SPAIR* Spectral Attenuated Inversion Recovery (Siemens Healthineers)

**Tab. 3:** Untersuchungsprotokolle für die Gelenkbildgebung bei 3 T aus dem Institut der Autoren

Beschleunigtes 3‑T-MRT-Protokoll des Schultergelenks mit DL-basierter Bildrekonstruktion					
Parameter	Axiale PD FS	Koronare T2 FS	Koronare PD	Sagittale T2 FS	Sagittale T1
Repetitions‑/Echozeit (ms)	4200/31	4350/54	3970/35	5030/53	786/11
PI-Faktor	3	3	3	3	3
SMS/FOV Shift Faktor	0/0	0/0	0/0	0/0	0/0
Echozuglänge	11	12	11	13	4
Receiver Bandbreite (Hz/Pixel)	260	252	279	217	279
Echoabstand (ms)	10,3	10,8	11,5	10,6	10,6
Field of view (mm)	140 × 140	140 × 140	140 × 140	140 × 140	140 × 140
Matrix	320 × 256	320 × 256	448 × 314	320 × 240	320 × 256
Schichtdicke (mm)	3	3	3	3	3
Schichtanzahl	30	28	28	28	28
Phasenkodierrichtung	Anterior-posterior	Kopf-Fuß	Kopf-Fuß	Kopf-Fuß	Kopf-Fuß
Phasen Oversampling (%)	70	120	120	120	120
Anzahl der Averages	1	1	1	1	1
Flip-Winkel (°)	150	150	150	150	150
Fettsättigung	SPAIR	SPAIR	–	SPAIR	–
Deep Resolve Sharp	On	On	On	On	On
Deep Resolve Boost	High	High	High	High	High
Akquisitionszeit (mm:ss)	01:09	01:18	01:32	01:20	01:20

*DL* Deep Learning,* PD* protonengewichtet, *PI* parallele Bildgebung, *FS* fettsupprimiert, *SMS* simultane Mehrschicht, *SPAIR* Spectral Attenuated Inversion Recovery (Siemens Healthineers)

**Abb. 5:**
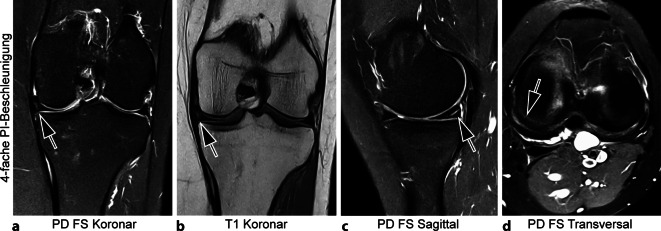
Vierfach PI-beschleunigte 0,55-T-MRT (Magnetresonanztomographie, parallele Bildgebung) des linken Kniegelenks mit Deep-Learning-Superresolution (DLSR; Gesamt-Akquisitionszeit: 09:48 min). 40-jährige Patientin mit mukoider Degeneration (*Pfeile* in **a** und **b**) und horizontalem Riss des Innenmeniskus (*Pfeile* in **c** und **d**)

Neben den bevorzugt in der muskuloskeletalen Diagnostik eingesetzten Feldstärken ≥ 1,5 T profitiert allerdings insbesondere auch die Niederfeld-MRT bei 0,55 T von der kombinierten Nutzung von Beschleunigung und DLSR-Rekonstruktion [15]. In der Bildgebung peripherer Gelenke kann so simultan eine Reduktion der Averages auf zwei und eine Erhöhung des PI-Faktors auf vier erfolgen. Die gesamte Akquisitionszeit einer Kniegelenk-MRT kann so bei guter Bildqualität auf unter 10 min verkürzt werden (Abb. 5). Gleiches gilt für die Bildgebung der Wirbelsäule. Ein vollständiges Untersuchungsprotokoll der Lendenwirbelsäule mit diagnostischer Bildqualität ist ebenfalls in einer Akquisitionszeit von 8 bis 9 min erreichbar und ermöglicht so eine nahezu zeitneutrale Nutzung der Niederfeld-MRT gegenüber der Bildgebung bei höheren Feldstärken.

## Fazit für die Praxis

- Die einzelne oder kombinierte Nutzung moderner Beschleunigungstechniken kann die reine Akquisitionszeit der muskuloskeletalen Magnetresonanztomographie (MRT) ohne Qualitätsverlust auf unter 10 min verkürzen.
- Deep-Learning-basierte Algorithmen bauen auf bestehende Bildrekonstruktionsmethoden auf und ermöglichen so die Nutzung höherer Beschleunigungsfaktoren.
- Die kombinierte Anwendung erlaubt die Akquisition diagnostischer muskuloskeletaler MRT-Untersuchungen je nach Untersuchungsregion in weniger als 5 min.
- Das Potenzial dieser Technologie ist noch nicht ausgeschöpft und wird in absehbarer Zeit die Untersuchungszeiten in der klinischen Praxis weiter verkürzen.

## Abkürzungen

CS: Compressed Sensing
DL: Deep Learning
DLSR: Deep Learning Super-Resolution
MRT: Magnetresonanztomographie
PI: Parallele Bildgebung
SMS: Simultane Mehrschicht
SNR: Signal-zu-Rausch-Verhältnis
TSE: Turbo-Spin-Echo
